# The softening of human bladder cancer cells happens at an early stage of the malignancy process

**DOI:** 10.3762/bjnano.5.52

**Published:** 2014-04-10

**Authors:** Jorge R Ramos, Joanna Pabijan, Ricardo Garcia, Malgorzata Lekka

**Affiliations:** 1 Instituto de Ciencia de Materiales de Madrid, CSIC, Sor Juana Inés de la Cruz 3, 28049 Madrid, Spain; 2 Centro de Estudios Avanzados de Cuba, Carretera de San Antonio de los Baños, km 1 ½, Valle Grande, La Habana, Cuba; 3 The Henryk Niewodniczański Institute of Nuclear Physics, Polish Academy of Sciences, Radzikowskiego 152, 31-342 Kraków, Poland

**Keywords:** actin filaments, atomic force microscopy (AFM), bladder cells, cytoskeleton, elastic properties of cells, malignancy degree of cancer cells

## Abstract

Various studies have demonstrated that alterations in the deformability of cancerous cells are strongly linked to the actin cytoskeleton. By using atomic force microscopy (AFM), it is possible to determine such changes in a quantitative way in order to distinguish cancerous from non-malignant cells. In the work presented here, the elastic properties of human bladder cells were determined by means of AFM. The measurements show that non-malignant bladder HCV29 cells are stiffer (higher Young’s modulus) than cancerous cells (HTB-9, HT1376, and T24 cell lines). However, independently of the histological grade of the studied bladder cancer cells, all cancerous cells possess a similar level of the deformability of about a few kilopascals, significantly lower than non-malignant cells. This underlines the diagnostic character of stiffness that can be used as a biomarker of bladder cancer. Similar stiffness levels, observed for cancerous cells, cannot be fully explained by the organization of the actin cytoskeleton since it is different in all malignant cells. Our results underline that it is neither the spatial organization of the actin filaments nor the presence of stress fibers, but the overall density and their 3D-organization in a probing volume play the dominant role in controlling the elastic response of the cancerous cell to an external force.

## Introduction

During oncogenic progression, many cancer-related alterations change both the internal structures of cells and also their surroundings, i.e., the extracellular matrix (ECM). During last two decades, much research has been carried out that demonstrated a larger deformability of living cancerous cells, which is in contradiction to the macroscopically detected stiffening of various tumors. Several papers demonstrated the possible influence of the ECM surrounding cancerous cells in the tumor progression [1–2]. The best example is breast cancer, whose solid tumors are detectable in macroscale by palpation whereas single cells show a larger deformability [3]. In this context, it has also been proposed that tumorigenesis in breast tissues is driven by changes in the mechanical properties of the extracellular matrix [2,4]. Remarkably, in this case the ECM of the malignant cells is stiffer (reflected by a higher Young’s modulus) as compared to the ECM of non-malignant tissues [2]. Those studies underline the connection between changes in the mechanical properties of the cell and the extracellular matrix, and the presence or development of progressive diseases. However, the specific role of the mechanical properties of the cells and the extracellular matrix in pathogenesis such as tumor progression remain poorly understood at best. The relationship between mechanics at the cellular level and tumorigenesis represents a new perspective for which many issues need to be addressed.

The studies of cell mechanics have gained great importance with the advent of AFM measurements, which demonstrated the capability to probe single cells and underlining a correlation between cell mechanics, in particular elasticity, and cancer [5–6]. The first measurements showed that cancerous human bladder cells were softer than non-malignant bladder cells. Further measurements of cancerous cells have confirmed that a lowering of the elastic modulus of the cells is a general feature of cancer transformation [6–12]. This change is probably associated with the enhanced capability to migrate and to adapt to changing environments, which is observed in metastasis. Importantly, these results are valid for other cell lines, such as ovary or prostate cancers [10], and can be extended also to primary cells [6,13] and tissue sections [5,12] that were collected from human patients. For example, a comparison of the elastic properties of normal and benign breast tissues gives a wide distribution of the Young’s modulus, in which a peak at lower values characterizes malignant tissues. It has also been found that the Young’s modulus distribution in cancerous cells is narrower than the distribution found in non-malignant cells [4,6]. However, the elements of the cell that contributes to its mechanical response as measured by the AFM need to be clarified.

AFM studies on cells are often combined with the use of drugs that modify the mechanical response of the cell [14–15]. The influence of the different elements of the cytoskeleton on the force response can be monitored by selectively inhibiting the formation of some of them [16–19]. By disaggregation of actin filaments with the use of different types of cytochalasin, Rotsch and Radmacher have reported a decrease of the Young’s modulus of fibroblasts [16]. Similar findings regarding the role of actin filaments have been reported in other types of cells such as lymphocyte and Jurkat cells [19]. The role of the microtubules (MT) in AFM measurements remains open. Pelling et al. performed immunofluorescence and AFM studies in order to determine the influence of the MT on the cell membrane in response to serum conditions and nocodazole [17]. Their results show that the stiffness depends on the interplay between dynamically different types of MT configurations (unstable and stable) and intermediate filaments (IF), which all act to impart a distinct cellular type of transient metastability. On the other hand, it has been reported that the disassembly of the MT of fibroblasts by using colchicine and colcemide does not lead to a softening of the cell [16]. The abovementioned research shows that disruption of cytoskeletal filaments is useful for assesing the link between the elastic properties of cells and the structure of the cytoskeleton. It is also worth mentioning that such an approach can be used for studying the influence of certain such compounds as chitosan [14] or cisplatin [20] on the mechanics of single cells, while pointing out that studies on the correlation between the mechanical properties and the structure of the actin cytoskeleton are particularly important for developing and monitoring cancer therapies.

It is well established that actin filaments are mostly responsible for the mechanical properties of cells that are measured by the AFM. Therefore, there have been several attempts trying to show the correlation between the 2D-organization of actin filaments and cells stiffness in relation to cancer invasion. The relation between the stiffness of cancer cells and the 2D-organization of the actin cytoskeleton has been reported for breast [3], thyroid [11] and ovarian [21] cancers. For stiffer cells, the actin filaments distribution usually revealed two types of filament organization, i.e., an actin cortex and well-formed linear actin bundles (i.e., stress fibers) that span over a whole cell. For softer cells, the actin filaments were less organized. Mostly, short filaments were observed. If F-actin bundles were present, they were shorter and randomly oriented. In particular for ovarian cancer, the correlation between the migratory behavior of cells and their stiffness has been demonstrated [21]. In other studies, the larger deformability of the mouse ovarian cells has been correlated with the dysregulation of actin (stress) fibers, which influences both fiber thickness and organization, as shown by confocal microscopy of early and late stages of ovarian cancer progression [22].

Here, we study the correlation between the elastic properties and the expression and organization of the actin cytoskeleton in human bladder cancer cells. We have chosen four cell lines with various histological grades. Those lines differ in terms of the organization of the cytoskeleton (presence/absence of stress fibers, distinct actin expression level) and the elastic properties measured by force spectroscopy. The cancerous cells are more compliant with respect to the non-malignant cells. Regardless of histological grades, the deformability of the cancerous cells reaches a similar level but the organization of actin filaments remains dependent on the cell type. These data underlines the complexity of the remodeling process in actin filaments in cancers that is expressed in the elastic properties of cells. Our findings show that in human bladder cancer cells, both the expression level of actin (in particular, β-actin) and its 3D-organization in the probing volume govern the elastic properties. The organization of actin filaments present on the surface of a cell as probed by AFM and fluorescence microscopy is not sufficient to fully explain the cellular stiffness. Additionally, the presence of actin fibers (stress fibers) is not a prerequisite for a deformability of cancer bladder cells. Moreover, the observed sudden drop of stiffness is a clear indication of cancer-related changes in human bladder cells, which strongly supports the usefulness of cell deformability in detecting cancer-related changes in bladder cancer. Such a relation has not been observed so far for any other cells measured by AFM in which usually a gradual drop of stiffness was observed.

## Results

### Cell morphology

**Figure 1:**
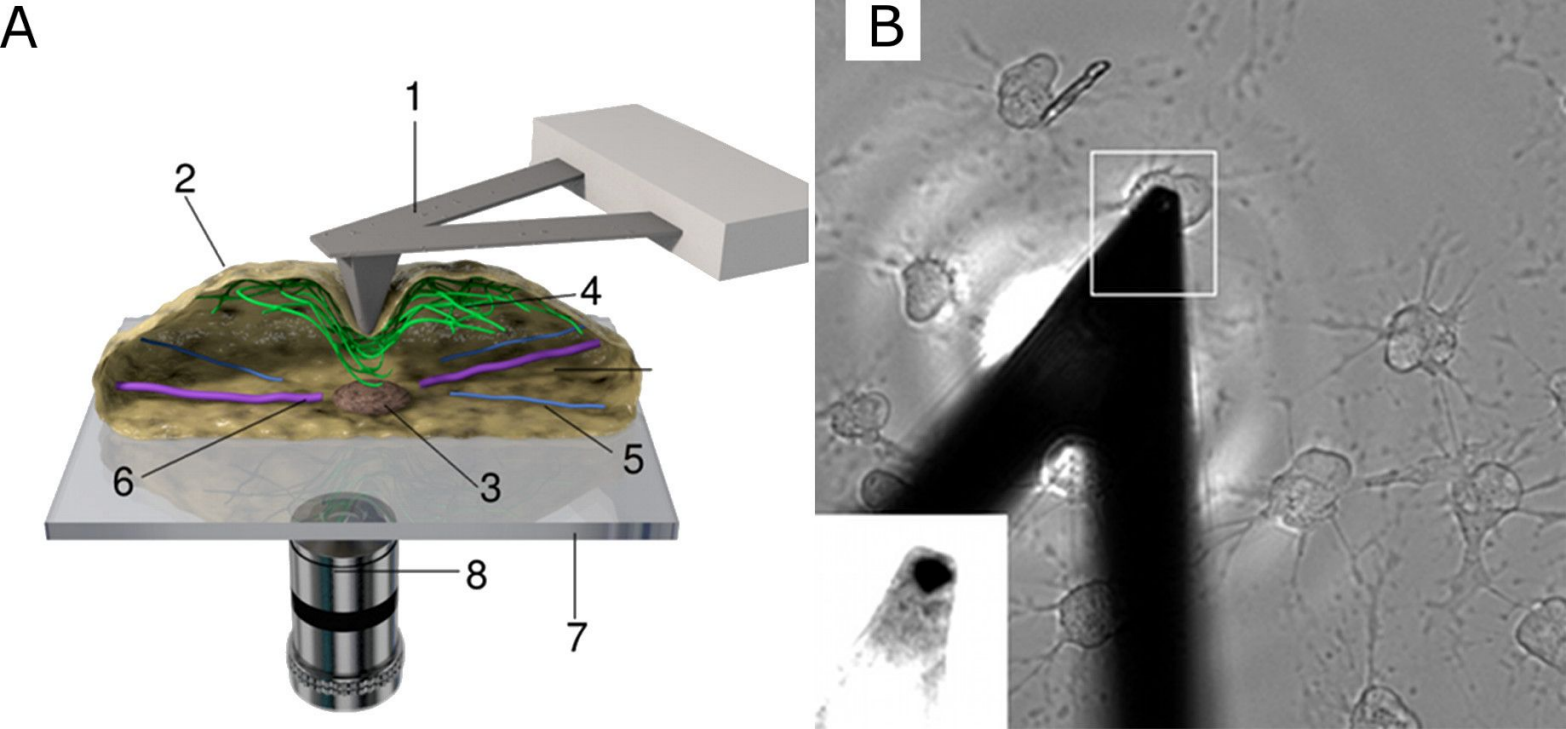
(A) Scheme of experimental setup. (1) V-shaped cantilever-tip ensemble; (2) cell membrane; (3) cell nucleus; (4) actin filaments; (5) intermediate filaments; (6) microtubules; (7) cover slip; (8) optical microscope lens. (B) Optical image of the cantilever positioned on top of the cell treated with cytochalasin D. After being treated with cytochalasin D, the cells shrink to a circular form.

Force spectroscopy experiments were performed on living cells, which were located on an inverted optical microscope (Figure 1). The optical microscope was also used to estimate the position of the AFM probe (tip) along the cell. Once a position was selected (close to the cell nuclei), the tip was approached towards the cell surface until a repulsive force reached the set point value of 100 pN.

**Figure 2:**
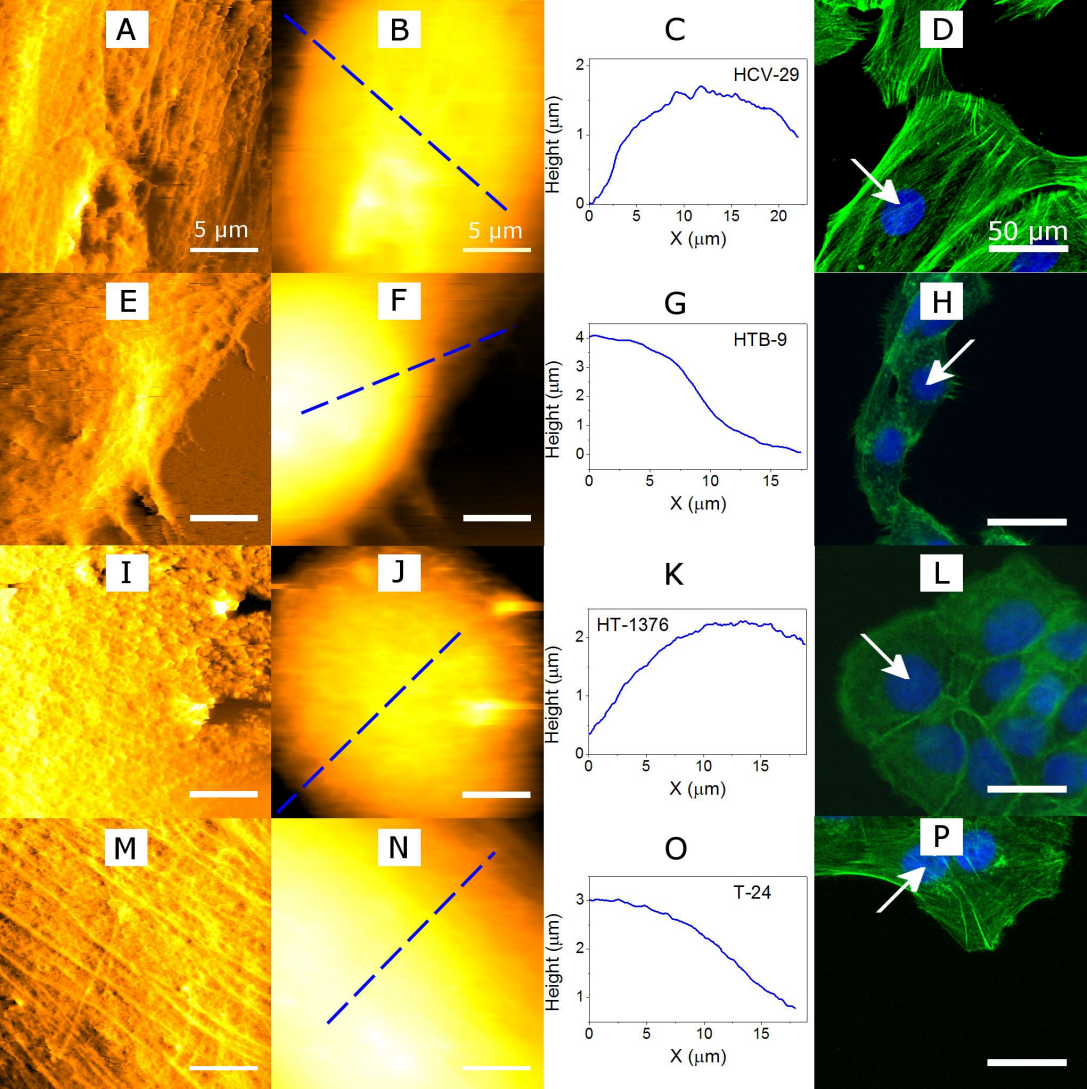
Force and fluorescence microscopy images of bladder cells. (A–D) Non-malignant cells, HCV29; (E–H), HTB-9 cells, grade 2; (I–L) HT-1376 cells, grade 3; (M–P) T24, grades 3 and 4. The AFM measurements were performed on regions centered over the cell nucleus marked with an arrow in the fluorescence images. The 1st column (panels A, E, I and M panels) is the AFM error signal. The 2nd column (B, F, J and N) shows the topography (AFM). The 3rd column (C, G, K and O) shows the apparent height profile. The 4th column (D, H, L and P) shows the fluorescence microscopy images. The arrows indicate the cells where the AFM images were recorded (blue: cell nucleus; green: actin cytoskeleton).

The AFM images were taken over an area of 20 × 20 μm^2^ with 512 pixels per line. The scan rate was varied from 0.5 to 1.0 Hz depending on the cell type. Figure 2 shows the AFM topography, error signal, single cross-sections and the fluorescence images of the cell lines studied here, non-malignant HCV29 and the malignant cells HTB-9, HT-1376, and T24, respectively. The AFM error image (panels A, E, I, and M) enables to visualize the cell cytoskeleton that lies beneath the cell membrane. The filaments observed by AFM correspond to the actin filaments. Although, they are dispersed throughout the whole cell, they are mainly concentrated close to the cell membrane to form the so called actin-cortex. The AFM error image shows that these filaments are organized in two groups: (i) short actin filaments and (ii) and bundles of long acting filaments (stress fibers). The presence of these two groups in the AFM image depends on the cell type. In the non-malignant HCV29 cells both short actin filaments and stress fibers are visible. Similarly, both structures are present in cancerous T24 cells (transitional cell carcinoma). However, the other types of malignant cells, HTB-9 (grade II, carcinoma) and HT-1376 (grade III, carcinoma) do not show the presence of stress fibers. These results are consistent with fluorescence microscopy images of actin filaments stained by using phalloidin that was labeled with Alexa Fluor 488 dye (Figure 2, panels D, H, L, P and Figure S1 in Supporting Information File 1). Similarly to the AFM images the stress fibers are only visible in the non-malignant HCV29 cells and the cancerous T24 cells.

The cross-sections along the marked lines on the AFM topography (Figure 2, panels C, G, K, and O) show the apparent height differences between two positions along the cell surface. One located above the nucleus and the other close to the cell edge. The height ranges from 3 to 4 μm depending on the cell type. The highest values were observed for HTB-9 cells (≈4 μm). The others cell types had similar height of about 3 μm. We note that the size of the nucleus does not seem to correlate with the observed height. For example, the HTB-9 cell has the smallest nucleus; however, it has the largest height as measured from the substrate baseline.

### Expression of actin

Actin is a globular protein that forms microfilaments. It exists in α, β, and γ isoforms. The α-actin was found in muscle tissues, the β- and γ-actins coexist in most cell types as components of the cytoskeleton [23–25]. Moreover, the transition from the epithelial to the mesenchymal phenotype reported for cancer progression requires a remodeling of the actin cytoskeleton [26]. This phenomenon is visible in the fluorescence and AFM images obtained for the four studied human bladder cell lines.

**Figure 3:**
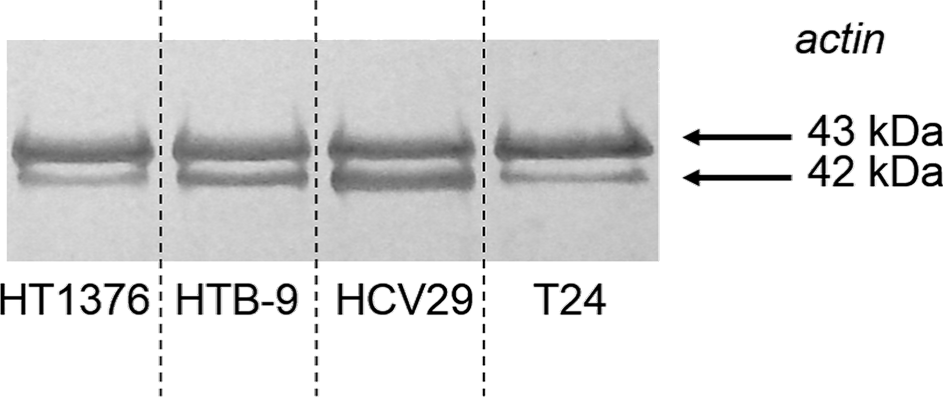
Total expression of actin in human bladder cells. The used antibody targets all known actin isoforms with a molecular mass of 42 kDa (globular actin and fibrous actin). The upper band (43 kDa) probably corresponds to cytoplasmic actin isomers with 43 kDa that possess the similar epitopes as those of 42 kDa.

In order to verify whether changes in the organization of the actin filaments are accompanied with the different expressions of actin, we have used the Western blot (Figure 3). The analysis was performed for the same number of cells (i.e., 2.56 × 10^6^ cells/mL) for each studied cell line. This analysis exhibited two bands at 42 and 43 kDa. The used antibody targets all the known actin isoforms with a molecular mass of 42 kDa in globular and fibrous forms. The band of 43 kDa probably corresponds to cytoplasmic actin isomers of 43 kDa that possess similar epitopes as those of 42 kDa.

The obtained results showed the higher amount of actin in non-malignant HCV29 cells and the lowest in cancerous HT-1376 (grade III, carcinoma) and T24 (transitional cell carcinoma) cells. To quantify the expression, the ImageJ program was used to determine the area under each band. The largest area corresponds to the HCV29 cells. The band area of the cancerous cells with respect to HCV29 was 73%, 35% and 42% for the HTB-9, HT-1376 and T24 cell lines, respectively. The results show that when cells with increasing tumor grading are measured, the amount of actin decreases in the cancerous cells as compared to non-malignant ones.

### Determination of the Young’s modulus

**Figure 4:**
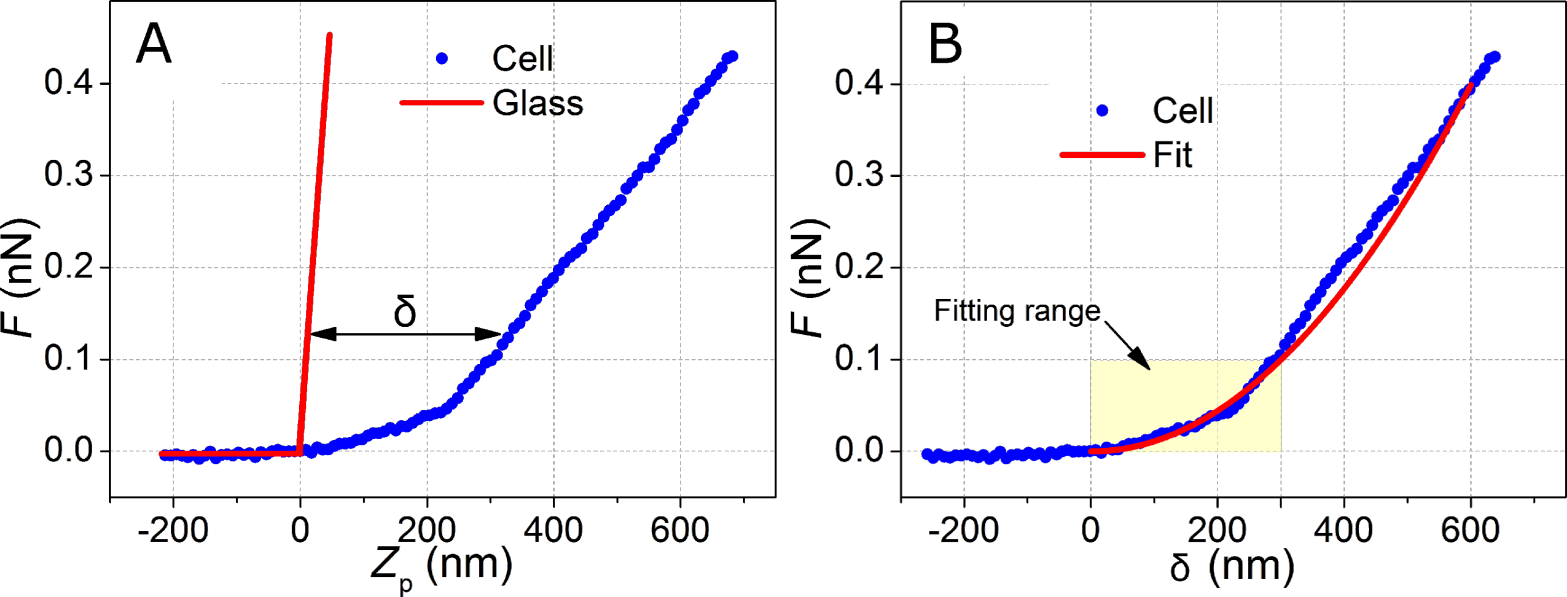
(A) Exemplary force curve recorded on a HT-1376 cell (blue dots). The red solid line represents a curve taken on a hard glass surface (calibration curve). The indentation δ is the horizontal difference between these curves (see Equation 2). The contact point is chosen as the origin. (B) Force-versus-indentation curve (blue dots) fitted with Sneddon’s equation for a conical tip (red line).

The elastic properties of the cells were determined by using Sneddon’s model for a conical shape of the tip [27]. First, the force curves were converted into force-versus-indentation curves and those curve were fitted to Equation 1 (see section Experimental: Force spectroscopy on living cells). Because the Young’s modulus of the tip is about 160 GPa (seven orders of magnitude larger than that of the cells), we can assume that Young’s modulus of the interface is *E*_eff_ ≈ *E*_cell_/(1 – ν^2^). Figure 4A shows a typical force curve obtained on a HT-1376 bladder cell together with a force curve obtained on a stiff surface (glass). For a given deflection (or force), the difference between the piezo displacement for the stiff and compliant surface gives the indentation (Equation 2, see section Experimental: Force spectroscopy on living cells). Figure 4B shows the force-versus-indentation curve derived from Figure 4A and the fit obtained with the Sneddon’s model assuming a conical AFM tip with half-angle of 20°. The Young’s modulus obtained from the fit was 6.1 kPa. The force spectroscopy data has been acquired in the region of the cell above the nucleus. The experimental force curves show some deviations from a parabola. Consequently, the parameters of the fitting will depend on the fitting length (indentation). For that reason, any conclusion derived from the data has to be verified for all the indentation depths studied here. On the other hand, the relative error in the determination of the contact point will decrease for larger indentations.

**Figure 5:**
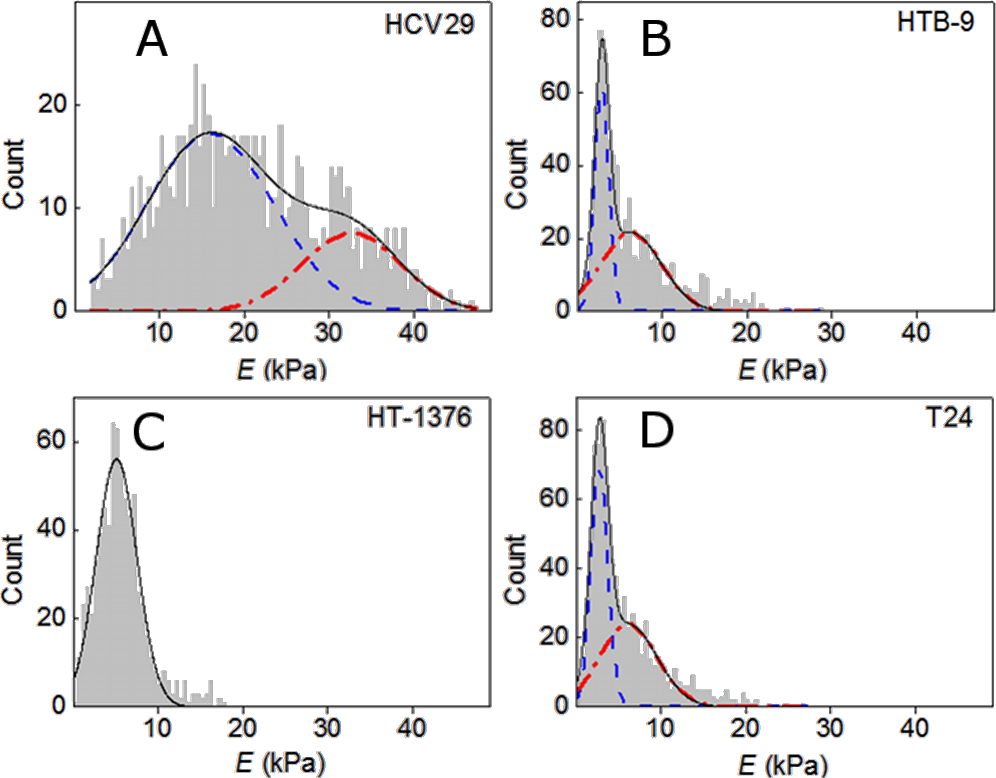
Distribution of Young’s moduli determined by fitting the curves within the indentation range up to 300 nm. The elasticity histogram is broader for the non-malignant cells (HCV29) than for the HTB-9 and the T24 cancerous cells. Histogram bin width of 500 Pa.

The results obtained for all recorded cell lines yielded the corresponding distributions of the Young’s moduli. Figure 5 shows the Young’s modulus values collected from the four bladder cell lines of this study and calculated for the indentation from 0 to 300 nm. Depending on the cell type distinct features are observed in the histograms. For the non-malignant cells (panel A), the observed distribution can be decomposed in two Gaussians, the larger one centered at 16.0 ± 0.9 kPa and the smaller centered at 33.0 ± 2.0 kPa. For HTB-9 (panel B) cells most of the data is centered at 3.0 ± 0.1 kPa with a small tail centered at 6.4 ± 0.5 kPa; for T24 (panel D) cells the dominant peak is centered at 2.9 ± 0.5 kPa while the secondary contribution has its maximum at 6.2 ± 0.6 kPa. The histogram for HT-1376 (panel C) cells shows a single maximum centered at 5.2 ± 0.1 kPa. We also observe that the histograms are narrower for all cancerous cells. This is in agreement with previous results that showed that the distribution of the Young’s modulus becomes narrower for metastatic cells [4,14].

Figure S2 (Supporting Information File 1) shows the Young’s moduli of the same cell lines, but in this case the fitting has been extended up to an indentation depth of δ = 500 nm. Two main conclusions are derived with respect to the data obtained at δ = 300 nm. First, the maxima of the distributions are shifted to lower values. Second, the distribution width is smaller. Those results apply for all bladder cell lines of this study.

### Depth-dependent changes of the Young’s modulus

**Figure 6:**
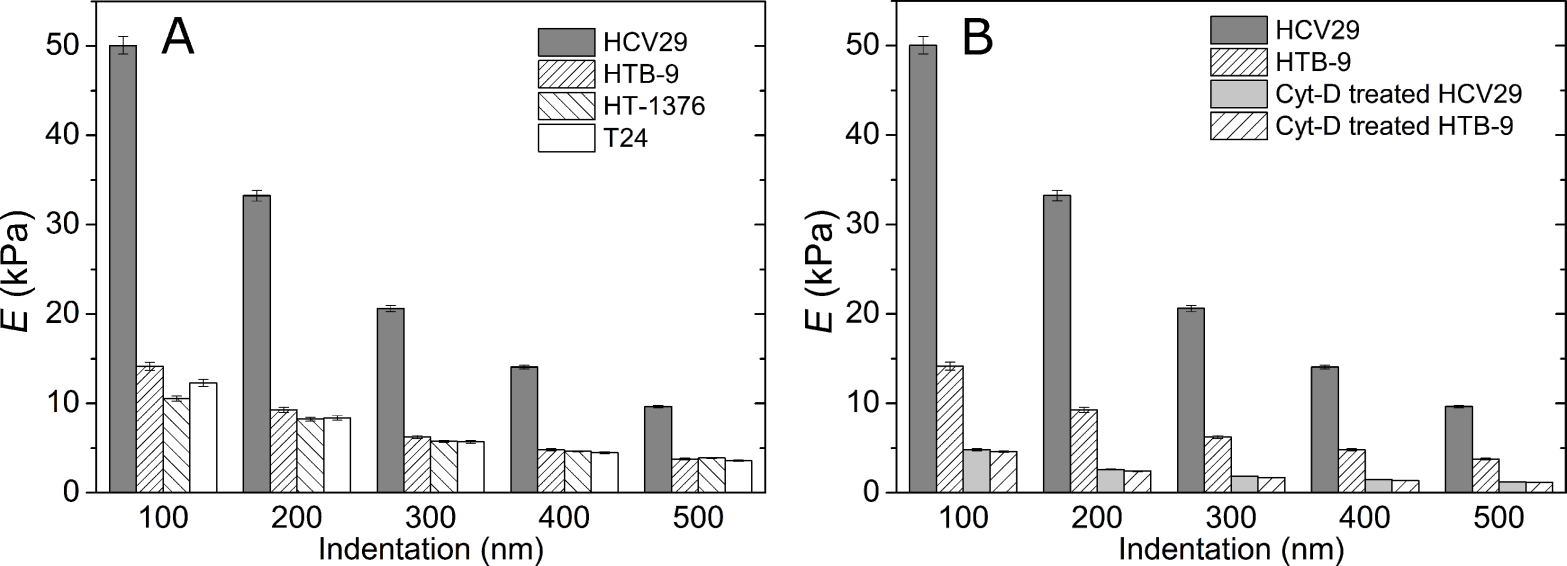
(A) Elasticity of living bladder cells as a function of the indentation depth. The data is presented as mean ± SD. (B) Comparison of the elasticity of the cells before and after treatment with cytochalasin D (Cyt-D). The data is presented as mean ± SD.

Figure 6A presents the changes of the Young’s modulus as a function of the indentation depth between 100 and 500 nm. For the non-malignant HCV29 cells the modulus goes from 50 kPa at 100 nm to 10 kPa at 500 nm. For the malignant cells the Young’s modulus decreases from about 15 kPa at 100 nm to 5 kPa at 500 nm. The decrease of the Young’s modulus with δ has been previously reported [18] although this effect is still not fully understood [5,18–19].

### Effect of cytochalasin D on the elasticity of the cells

In order to verify the influence of the actin filaments and their structure on *E*_cell_, some of the cell lines were treated with cytochalasin D (Cyt-D), which is known to depolymerize the actin filaments. This results in a reduction of the Young’s modulus [16]. However, Cyt-D does not affect the structure of MT or IF. Figure S3 (Supporting Information File 1) shows the Young’s moduli of the HCV29 cell line before and after exposure to Cyt-D measured at δ = 300 nm. The actin cytoskeleton of HCV29 cells is composed of short actin filaments and stress fibers. The stress fibers are visible in Figure 2 and Figure S1 (Supporting Information File 1) for the cell lines HCV29 and T24. After adding Cyt-D, the modulus drops from 21 to 2 kPa. We note that the differences between treated and non-treated cells are preserved across the different individual cells and cell locations. The Young’s moduli measured on different cells of the same cell line are similar (Figure S3, Supporting Information File 1). In fact the variability observed for each cell type could be attributed to local changes in the region around the cell where the measurement has been performed. The measurement in each of the panels of Supplementary Figure S3 has taken over 4 h.

The actin cytoskeleton structure of HTB-9 cells is mainly composed of short actin filaments. In these cells, there are virtually no stress fibers to be seen. In this cell line a treatment with Cyt-D causes a decrease of *E*_cell_ from 15 kPa to 5 kPa (δ = 100 nm) and from 6 kPa to 2 kPa (δ = 300 nm). Figure 6B shows the change of the Young’s moduli for HCV29 and HTB-9 cells before and after exposure to Cyt-D for different indentations. Interestingly, the elastic response of non-malignant and malignant cells is almost identical after exposure to Cyt-D.

### Statistical significance of the cells Young’s modulus

The statistical significance of the Young’s modulus measurements for the different cell lines was verified by applying the Tukey–Kramer method [28]. Here the test was performed at a significance level of *P* = 0.05 (Table 1). The test shows statistically significant differences between the Young’s modulus values of HCV29 non-malignant cells and those obtained on cancerous cells. Similarly, relevant statistical differences are found between the values measured before and after the treatment with cytochalasin D. It also shows that among cancerous cells (HTB-9, HT-1376 and T24) there are no statistical differences in their values, at least for indentation depths between 200 and 400 nm. It is worth to mention that cells treated with Cyt-D had similar Young’s moduli for both cell lines (HCV29 and HTB-9).

**Table 1:** Tukey–Kramer multiple comparison test of the results shown in Figure 6 with significance level *P* = 0.05 for all indentation depths.^a^

cell type	HTB-9	HT-1376	T24	HCV29 + Cyt-D	HTB-9 + Cyt-D

HCV29	***	***	***	***	***
HTB-9		ns	ns^b^	***	***
HT-1376			ns^b^	***	***
T24				***	***
HCV29 + Cyt-D					ns

^a^*** *P* < 0.001; ns: no significant differences between samples; ^b^There are two exceptions for the data represented: for HTB-9–T24 at 100 nm *P* < 0.01 and for HT-1376–T24 at 500 nm *P* < 0.05.

## Discussion

The integration of force and fluorescence microscopies enables one to obtain comprehensive information about the morphology of the cells. The latter provides the information about fluorescently labeled structures while AFM delivers the topology and the mechanical properties of the sample. In our studies, the fluorescent images of actin filaments were compared with the surface topography. The cytoskeleton is important for a normal cell function, however, cancer progression changes its role by using it as a tool to alter cell growth, stiffness, movement and invasiveness [26]. The actin cytoskeleton serves as a scaffold for signaling, as a connection to the extracellular environment, and as a mechanosensor. However, there is no general evidence that changes in the expression or organization of actin promotes or inhibits cancer metastasis. It seems that cancerous cells reorganize the actin cytoskeleton to alter the growth or adhesion or the mechanical properties to enhance their survival rate during various phases of tumor progression and metastatic spreading. This reorganization process involves both re-arrangements of actin filaments in the cell and also changes in the actin expression [27]. Our results on human bladder cancer cells showed that the level of actin (a component of the cell cytoskeleton and also a mediator of internal cell motility) in non-malignant HCV29 cells is the highest. Its drops as follows: HTB-9 (grade II, carcinoma) > T24 (transitional cell carcinoma) > HT-1376 (grade III, carcinoma) cells. Moreover, in non-muscles cells, the actin cytoskeleton in cells can be divided into two groups: short actin filaments forming a cortex and long stress fibers that consist of bundles of single actin filaments. The actin cortex is located beneath the cell membrane while the actin stress fibers span over the whole cell. The results obtained on human bladder cells (non-malignant HCV29 and three cancerous lines HTB-9, HT-1376 and T24) showed various organizations of the actin cytoskeleton as observed by fluorescence microscopy. In non-malignant HCV29 cells, both short actin filaments and stress fibers are visible. Similarly, both structures are present in cancerous T24 cells (transitional cell carcinoma). The two other cancerous cells, HTB-9 cells (grade II, carcinoma) and HT-1376 (grade III, carcinoma) only show the presence of short actin filaments. In recent decades, a novel functionality of the AFM, i.e., stiffness tomography, has been demonstrated [29–30] for neurons to deliver a 3D recording of the Young’s modulus. The results have shown the presence of hard structures, which are attributed to cortical actin cytoskeleton. However, the organization and mechanical properties of single actin filaments stress fibers have not been shown by this technique. Nevertheless, the imaging of the cell surface by using AFM in contact mode can convey information about the superficial layers of the actin cytoskeleton. In our case the topography of each studied cell lines correlates with the images of phalloidin stained actin filaments, which were recorded by using fluorescence microscopy.

The actin cytoskeleton has been reported to play the main role in the mechanical properties of living cells [15,31]. The observed increase of cellular deformability, which is induced by cytochalasin D, relates to the mechanical properties of the cytoskeleton with the actin network lying beneath the cell membrane. However, it cannot be specified, which form of spatial organization of actin filaments dominates. The highest expression of actin and the most abundant presence of stress fibers, which is observed in HCV29 cells, were accompanied by a large value of the Young’s modulus. It is clearly visible that a lower expression of actin, which is observed in malignant cells (HTB-9, HT-1376 and T24), is associated with a lower Young’s modulus. All malignant cells showed similar values of the Young’s modulus. However, the elastic properties of the cancerous cells seem to be independent of the presence/absence of stress fibers. The stress fibers are present in T24 cancerous cells (transitional cell carcinoma) but not in HTB-9 (grade II, carcinoma) and the HT-1376 (grade III, carcinoma).

The altered elastic properties of single cells is nowadays almost believed to be a general feature of cancer progression, which has been already shown in various measurements performed by using atomic force microscopy. The examples encompass human bladder [5,32], breast [3], colon [31], prostate [10,32], thyroid [11] and cervical cells [33]. A similar relationship was obtained in previous studies for human bladder cancer cells [5]. The non-malignant HCV29 cells were significantly stiffer than the cancerous ones. It should be also pointed out that these measurements are in agreement with the data reported earlier, in which the stiffness of both HCV29 and T24 cells was measured by force spectroscopy [5,14]. This observation is particularly important since these cells were grown previously on a glass coverslip covered with poly-L-lysine. In the present studies no glass surface modification was introduced. Thus, substrate chemical properties do not change the relationship between non-malignant HCV29 and cancerous T24 cells. The comparison performed in the previous studies showed that two non-malignant cell lines (HCV29 and Hu609) were significantly stiffer than three cancerous ones, i.e., Hu456 (grade I, carcinoma), T24 (transitional cell carcinoma), BC3726 (HCV29 cells transformed with ν*-ras* oncogene). However, in the studies from 1999 [5], there was no fluorescence microscopy involved to show the organization of actin filaments in the studied cell lines. In our work we have observed the same relation. Independently of the histological grade, the cancerous cells are more deformable. This point out the usefulness of the AFM to detect bladder cancer cells –regardless of the state of cancer progression– soft cells indicate a malignant phenotype.

The fluorescent images of the actin filaments, obtained in the present study, showed surprisingly that the 2D-organization of these filaments on the cell surface is not solely responsible for the observed stiffness of all malignant cells. It seems that in our case, the elastic properties are governed by both the expression level of F-actin and its 3D-organization in the probing volume.

The dependence of the Young’s modulus on the indentation depth represents another important element of the presented data. In the range studied here between 100 and 500 nm, the Young’s modulus decreases with the indentation depth (Figure 6A). The decrease of the Young’s modulus with the indentation depth indicates little influence of the solid support on the measurements [34]. Here we are focused on the ability to distinguish cells of different malignancy degrees. Consequently, in order to visualize the variations across the different cells lines we have normalized the Young’s modulus of the different cells to the one obtained at the same indentation for the non-malignant cells. Two main observations are obtained from the normalized plot. First, non-malignant bladder cells are stiffer than malignant cells. Second, for the cell lines studied here, the histologically determined tumor grade does not seem to be reflected in the elastic properties of the cells.

The treatment of cells with cytochalasin D resulted in a marked decrease of their Young’s modulus (Figure 6B). The Cyt-D inhibits the actin polymerization by binding to the fast growing plus ends of the microfilaments and blocking both the assembly and the disassembly of individual actin monomers from the bound end. It has been reported that the results of its action is a drop of the overall cell elasticity [16,33]. The correlation between the reduction of the Young’s modulus and the depolymerization of the actin filaments supports a predominant role of these filaments on the stiffness of the cells. In order to verify how the organization of the actin cytoskeleton influences the mechanical properties, HCV29 and HTB-9 cells were treated with 5 μM Cyt-D. The observed decrease of the elastic modulus confirms the pre-dominant influence of actin filaments on the elastic properties. The results was independent of both the 2D organization of actin filaments and on the expression level of actin (similar values were obtained 1.8 ± 0.1 kPa and 1.7 ± 0.1 kPa for HCV29 and HTB-9, respectively). The deformability of all studied cancerous cells is slightly larger than that of the cell treated with cytochalasin D. This points out to partial but extensive changes in actin cytoskeleton structure induced by cancer progression.

The relation between the cell deformability and the degree of invasiveness has attracted attention almost from the beginning of the elasticity measurements of cancerous cells. The comparison between various cell lines of the same origin like breast, prostate, bladder, thyroid, and ovarian cancers, only shows such a relation for ovarian cancers [21]. In the other research either only a comparison between only two cell lines has been done or such correlation could not be shown clearly. In our case, for bladder cancer, the first results from 1999 showed a significant difference between non-malignant and cancerous cells without any consideration of their degree of invasiveness. The results presented here, now clearly demonstrate that most probably, in human bladder cancers, cells acquiring a more invasive phenotype become more deformable at early stages of cancer progression. This makes the cell stiffness a powerful biomarker for detecting cancer-related alterations in human bladder tumors.

## Conclusion

Nanomechanical measurements performed with a force microscope at the single cell level have been applied to characterize the elastic properties of human bladder cancer cells. All malignant bladder cells have Young’s moduli about 2–3 times lower that those non-malignant cells. The low Young’s modulus (higher cellular deformability) seems to occur at an earlier stage of cancer progression and it does not seem to evolve with the metastatic phenotype. It correlates with a partial lack and/or depolymerization of the actin filaments. The data implies that the elastic response is dominated by the expression of F-actin. However, the presence of actin stress fibers (observed in non-malignant HCV29 and cancerous T24 cells) is not a prerequisite for a smaller cellular deformability of cancer bladder cells. The stress fibers observed in T24 cells produce a rather small increase of the Young’s modulus. This strongly suggests that the deformability of cancer cells is mainly related to the 3D-organization of actin filaments (both short filaments, and stress fibers) together with their density. The elastic properties of cancerous cells (HTB-9, HT-1376 and T24) were independent of the cell line, the stage of cancer progression, and the cell cycle. Therefore, they underline the usefulness of atomic force microscopy to detect bladder cancer cells because regardless on the state of cancer progression, softer cells indicate a malignant phenotype.

## Experimental

**Cell lines:** Four bladder cell lines with epithelial origin have been studied: non-malignant transitional epithelial cells of the ureter (HCV29, Institute of Experimental Therapy, Wroclaw, Poland), which serve as a reference; urinary bladder cell carcinoma (HTB-9, grade II, ATCC, LGC Standards); urinary bladder cell carcinoma (HT-1376, grade III, ATCC, LGC Standards) and transitional cell carcinoma (T24, ATCC, LGC Standards).

The HCV29 and the T24 cells were grown in RPMI-1640 medium (Sigma) supplemented with 10% Fetal Bovine Serum (FBS, Sigma). The HTB-9 cells were grown in RPMI-1640 supplemented with 10% FBS, 1% HEPES (4-(2-hydroxyethyl)-1-piperazineethanesulfonic acid, Sigma) and 1% sodium pyruvate (Sigma). Finally, the HT-1376 cells were grown in Eagle’s medium (EMEM, LGC Standards) supplemented with 10% FBS (LGC Standards). The cells were cultured on glass coverslips placed inside plastic Petri dishes at 37 °C in a 95% air/5% CO_2_ atmosphere. The relative humidity was kept above 98%. The cells were measured 3 to 4 days after seeding. The cells treated with cytochalasin D (Sigma) were first rinsed with RPMI-1640 medium and then they were immersed in a 5 µM Cyt-D solution in RPMI-1640 medium for 1 h at 37 °C.

**Actin expression in bladder cells:** The expression of β-actin in the studied human bladder cells was determined by using Western blot analysis, performed in analogous way as in [35]. Briefly, for this analytical technique, which uses gel electrophoresis to separate native proteins, the same number of cells of about 2.56 × 10^6^ cells per mL for each cell line was lysed and loaded on 12% gels for SDS-PAGE and afterwards transferred to a polyvinylidene difluoride membrane (Roche). After blocking with casein solution, the membranes were then incubated overnight with the primary antibody against actin (1:10000, 0.025 µg/mL, BD Biosciences).

**Force spectroscopy of living cells:** Force spectroscopy measurements were performed by using a commercial AFM (model XE120, Park Systems, South Korea). We used v-shaped silicon nitride cantilevers terminated with a silicon tip (MLCT-C, Bruker, USA). Those cantilevers are characterized by a nominal spring constant *k* = 0.01 N/m while the length of the tip length is about 3 µm long, with a half-angle of 20° and a radius of 20 nm. The sensitivity of the photodiode was calibrated on a rigid glass surface.

Force-versus-distance curves (force curves hereafter) were acquired in a 25-µm^2^ region centered above the nucleus of the cell. The curves were acquired in 64 different positions separated by about 600 nm. The maximum indentation depth was established by the value of the peak force; in this case, it was set to 1 nN. The curves were acquired with a speed of 5 μm/s. The force-versus-indentation curves were obtained by subtracting the force curves recorded on a stiff glass surface from the ones recorded on the cell surfaces. The elastic modulus (Young’s modulus) was determined by fitting the force-indentation curve (approach section) to the Sneddon’s model [27,36] while assuming a conical tip. Then, the indentation depth and the effective Young’s modulus *E*_eff_ are related by

[1]
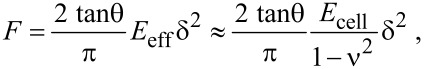


where *F* is the loading force, θ is the half-angle of the cone, ν is the Poisson coefficient of the cells (0.5 in this study), and δ is the indentation depth. The indentation is determined from the displacement *z_p_* of the piezo-scanner, the initial contact distance *z_0_*, and the deflection given by a hard wall *F/k*,

[2]
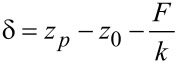


All experiments were performed at room temperature in a culture medium RPMI-1640 supplemented with 10 mM HEPES. For the cells treated with Cyt-D the buffer was 5 μM Cyt-D in RPMI-1640 medium.

**Statistical analysis:** We have analyzed 10 to 16 cells for each cell line (about 700 individual force curves per cell line). The Tukey–Kramer test (*P* = 0.05) was used to confirm statistical differences between Young’s modulus of different cell lines.

**Fluorescence microscopy:** The cells grown on a coverslip were washed with a RPMI-1640 medium supplemented with 10 mM HEPES. Next the cells were fixed by adding a 3.7% paraformaldehyde solution in PBS (phosphate buffered saline, Sigma) for 10 min and then washed with PBS buffer. The cell membrane was permeabilized by incubating the cells with a 0.2% Triton X-100 (Sigma) in PBS solution for 5 min. Finally, cells were rinsed with PBS buffer. Such prepared coverslips with cells were fluorescently labeled. The actin filaments were stained with phalloidin fluorescently labeled with Alexa Fluor 488 (Invitrogen) dissolved in PBS buffer (1:200) for 30 min. Then, the sample was washed with PBS buffer and the cell nuclei were stained with Hoechst stain (Invitrogen) in PBS buffer (1:5) for 15 min. The coverslips containing the cells were washed with PBS buffer, sealed using nail polish, and stored at 4 °C in the dark for 24 to 48 h before image recording.

**Force and fluorescence microscopy imaging of the cells:** The AFM is integrated with the inverted optical microscope equipped with fluorescent functionality. This enables to image the same region with both techniques and to correlate AFM and fluorescence data. The fluorescence microscopy was performed by using an Olympus IX71 inverted microscope (Olympus, Japan) equipped with a 100 W mercury lamp and U-MWIB2 and U-MWIG2 filters used for actin filaments (Alexa Fluor 488) and cell nuclei (Hoechst stain) visualization, respectively. For image recording, the Olympus XC10 digital camera (resolution 1376 × 1032 pixels) was used. Images were recorded using the program CellSens Dimension (Olympus). The AFM is equipped with a liquid cell sitting on a *x*–*y* piezoscanner with a range of 100 × 100 µm^2^. The approach and retraction of the AFM probe is realized using a separate piezoscanner with a *z*-range of 10 μm. The AFM images were obtained in contact mode by applying a maximum force of 100 pN. The imaging scan line rate was between 0.5 to 1.0 Hz with a length between 20 to 50 μm. Each image has 512 × 512 pixels.

**Western blot method:** The Western blot technique is a standard technique used in cell biology to detect specific proteins in the sample. It follows through two steps. First, an electrophoresis is performed to separate proteins from a cell extract. Second, proteins are transferred to a membrane, on which they are stained using monoclonal antibodies. Each band correspond to a specific protein with a particular molecular mass.

## Supporting Information

File 1 Additional experimental details.
