# Supplementary material for: The softening of human bladder cancer cells happens at an early stage of the malignancy process
Source: Beilstein J Nanotechnol. 2014 Apr 10;5:447–57. doi: 10.3762/bjnano.5.52 (PMC3999871; doi:10.3762/bjnano.5.52)
Supplement: File 1 — Additional experimental details. [file Beilstein_J_Nanotechnol-05-447-s001.pdf]

Supporting Information

for

The softening of human bladder cancer cells happens at an early stage of the malignancy process

Jorge R. Ramos[1,3], Joanna Pabijan[2], Ricardo Garcia*[1] and Malgorzata Lekka*[2]

Address: [1]Instituto de Ciencia de Materiales de Madrid, CSIC, Sor Juana Inés de la Cruz 3, 28049 Madrid, Spain; [2]The Henryk Niewodniczański Institute of Nuclear Physics, Polish Academy of Sciences, Radzikowskiego 152, 31-342 Kraków, Poland and [3]present address: Centro de Estudios Avanzados de Cuba, Carretera de San Antonio de los Baños, km 1 ½, Valle Grande, La Habana, Cuba.

E-mail: Ricardo Garcia* - r.garcia@csic.es;

Malgorzata Lekka* - Malgorzata.Lekka@ifj.edu.pl

* Corresponding autor

Additional experimental data

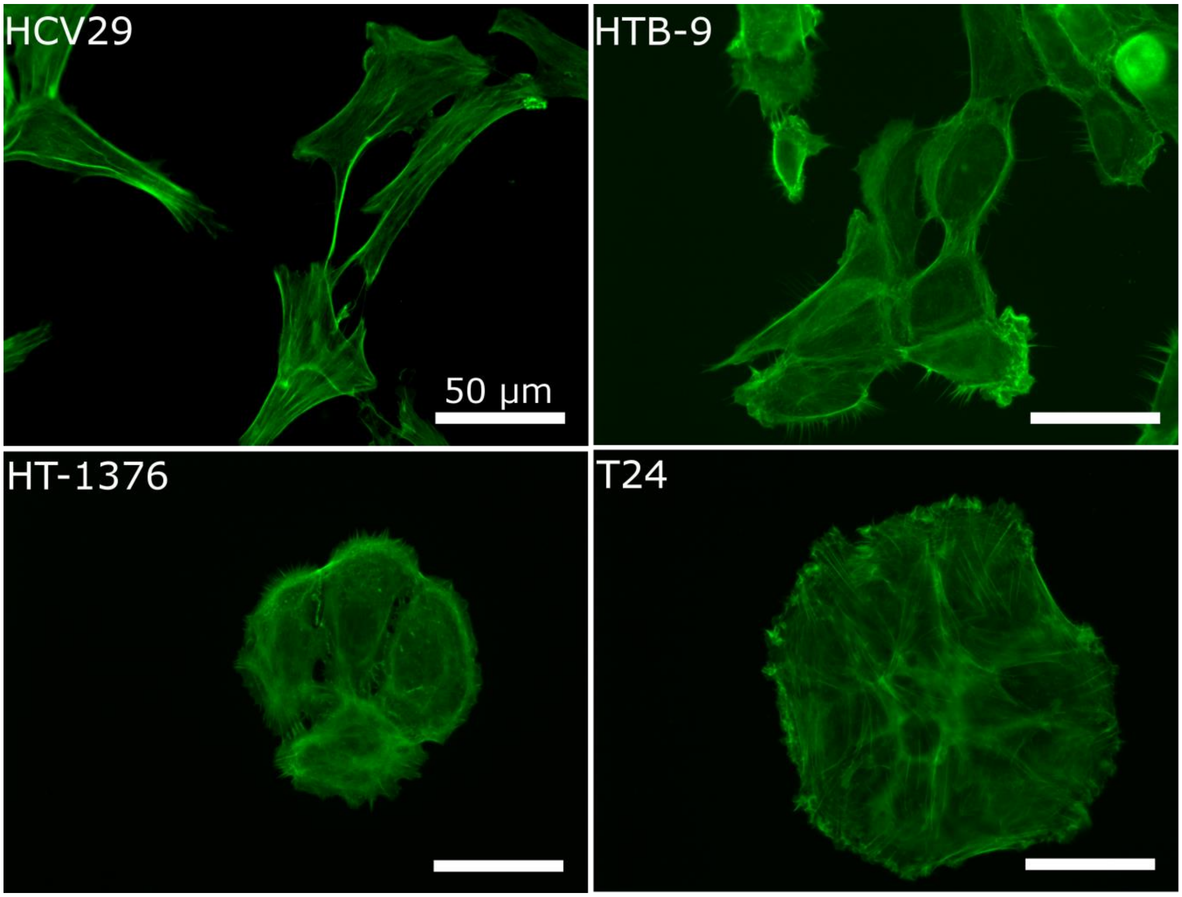

Figure S1: Organization of actin filaments in human bladder cells. The stress fibers are visible in HCV29 and T24 cells.

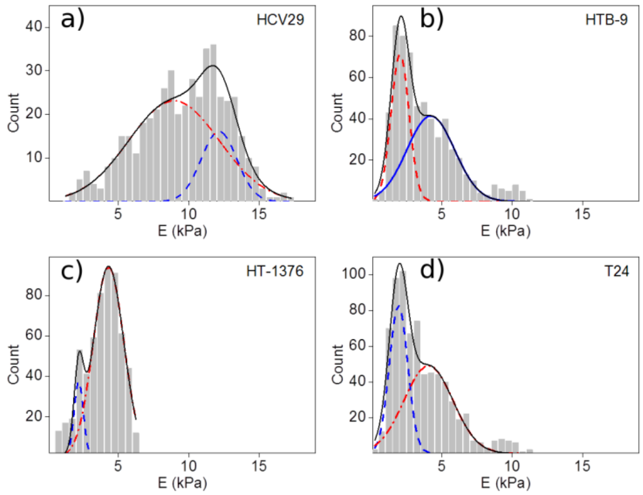

Figure S2: Distribution of the Young's moduli obtained by fitting the curves up to an indentation depth of 500 nm.

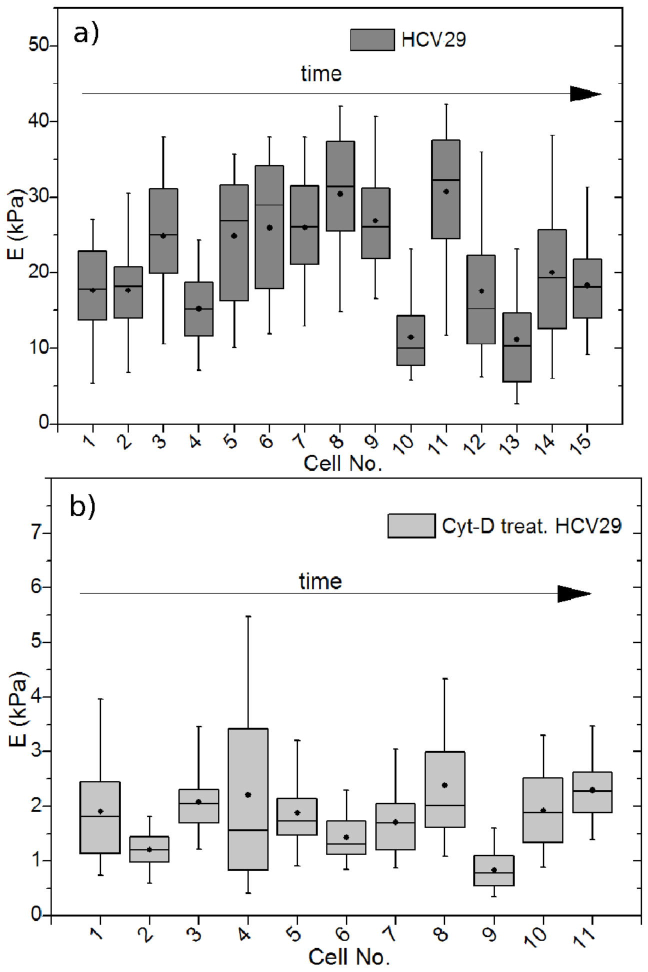

Figure S3: Effect of the exposure to Cyt-D on the cell elasticity. (a) Untreated HCV29 non-malignant cells. (b) HCV29 cells treated with Cyt-D. The dot in the box chart represents the mean value. The box boundaries are the 25, 75 percentiles. The line inside the box is the 50 percentile (median) while the whiskers are the 5 and 95 percentiles. For each cell line the measurements were taken in a single session lasting 4 h (room temperature). Fitting performed at δ = 300 nm. Please note different scales in Y-axis in A and B.
